# Quality Assessment of YouTube Videos As Information Source for Breast Self-Examination

**DOI:** 10.7759/cureus.70227

**Published:** 2024-09-26

**Authors:** Mohammed S Bu Bshait, Abdullah Almaqhawi

**Affiliations:** 1 Department of Surgery, King Faisal University, Al Ahsa, SAU; 2 Department of Family and Community Medicine, King Faisal University, Al Ahsa, SAU

**Keywords:** breast cancer, breast examination, education, video, youtube

## Abstract

Introduction

Breast self-examination (BSE) is essential for early detection of breast cancer to lower the disease's morbidity and death rate. Education about the proper application reinforces its effectiveness. YouTube is an emerging modality for education distribution. Thus, we aimed to evaluate the quality and reliability of BSE videos on YouTube.

Materials and methods

A web search of YouTube was conducted using the term "breast self-examination". The first 50 relevant videos found through this search were compiled and evaluated. Video reliability was evaluated by applying benchmark criteria from the Journal of the American Medical Association (JAMA). The educational quality of the videos was evaluated using the Global Quality Score (GQS) and the guidelines' comprehensiveness score for BSE-specific instructions.

Results

The mean number of views was 311,625.9. Medical sources were the most common upload sources, which were found in 60% of the analyzed videos (30 videos), while examination demonstration was the most common type of video content (33 videos, 66%), followed by examination information (15 videos, 30%). However, a significant association was found between videos containing both examination information and demonstration and better educational quality. Regarding video reliability, 34% of videos (17 videos) scored 0, and only 2% (one video) scored four. According to the GQS, only 8% (four videos) were of excellent quality, while the majority (20 videos, 40%) were of suboptimal quality. Based on the BSE comprehensiveness score, the mean score was seven out of nine.

Conclusions

Videos containing examination information and demonstrations showed the best educational quality. Although most of the YouTube videos of BSE showed a high comprehensiveness score for BSE-specific instructions, their JAMA reliability and GQS scores were poor.

## Introduction

Breast cancer is a major health concern worldwide, with rising incidence and mortality rates predicted in the coming years [1]. It is the most common cancer diagnosed globally in women, and with many new cases and associated deaths annually, it presents a significant public health challenge [2-4]. Breast cancer has a particularly severe impact on low- and middle-income countries, where access to high-quality care and difficulties in early detection lead to worse outcomes [5-7]. Breast cancer is predicted to account for 2.3 million cases and 685,000 deaths from cancer globally in 2020 [8,9]. In 2020, breast cancer ranked first for both incidence and mortality in the majority of countries, accounting for approximately 24.5% of all cancer cases and 15.5% of cancer deaths among women [8]. Breast self-examination (BSE), which helps women become more acquainted with their breasts and equips them to recognize any changes quickly, is a crucial practice in raising awareness of breast cancer and assisting in its early detection [10]. BSE is essential for early detection of breast cancer to lower the disease's morbidity and death rate [11]. The main objective of BSE is to enable women to recognize any potential changes in their breast topography by educating them about the typical topography [12]. Monthly BSE is recommended as a key component for detecting breast cancer at an early stage [13]. BSE is an easy and affordable way for women to monitor changes in their breasts and obtain medical help as soon as any abnormalities are noticed [14,15]. Women who are encouraged to undergo breast cancer examinations (BSE) are more "breast aware", which increases the likelihood of an early diagnosis and increases survival rates [12,16,17].

The internet is essential for distributing educational materials, enabling practical learning, and encouraging knowledge exchanges across a range of demographics [18-20]. Individuals can participate in learning activities and access educational resources on a flexible and accessible platform through online education [21,22]. Additionally, online education can improve preparedness, encourage community engagement, and increase resilience to disasters by implementing gamified educational initiatives [23]. The internet can be used as a source of patient education; for example, YouTube provides several significant benefits to the public. Patients can find various educational videos on YouTube, including those about medical conditions, treatment options, and healthcare practices [24,25]. Individuals can learn from their homes about their medical conditions, available treatments, and preventive measures [24,25]. The online resource provides patients with useful information and tools to improve their health literacy by offering a wide selection of instructional videos on medical procedures, diseases, and treatments [26,27]. Hence, the educational potential of YouTube videos can be integrated into formal educational settings for learning enhancement [28]. The importance of accessible educational tools, like BSE videos, in increasing breast cancer awareness has been highlighted alongside the rising engagement with digital content. Moreover, the evolving role of breast self-examination and how videos can play a critical role in teaching the proper technique and ultimately improving both the accuracy and frequency of self-examinations have been reported [29]. However, inaccurate information could be distributed through internet channels. Furthermore, it is crucial that viewers of these videos receive the most accurate information to detect any abnormality early and prevent it from worsening.

It is well-established that breast cancer has a significant global burden, emphasizing the importance of ongoing research, early detection, and effective treatment strategies to address this prevalent and devastating disease. Therefore, this study aims to determine whether YouTube is a reliable source of medical information for patients and evaluate the instructional quality and dependability of publicly accessible YouTube videos on self-breast examination.

## Materials and methods

Using the methodology used in earlier studies on related health conditions [30-32], the term "breast self-examination" was entered into the search bar of YouTube (YouTube LLC, San Bruno, CA, USA) on 11/03/2024. The videos were sorted according to their relevance with no additional filters. The first 50 relevant videos found through this search were compiled and evaluated. The study excluded videos that contained advertisements, were 30 seconds or less in length, repeated twice or more, or were in a language other than English from the collection of videos that were viewed. The subsequent video was included in cases where a non-relevant or ineligible video was excluded. The titles of the videos that satisfied the requirements for inclusion were noted.

Video characteristics

Consistent with earlier studies [33], the subsequent video attributes were noted for every eligible and featured video: first, the title; second, the length of the video; third, the number of views; fourth, the type of content; fifth, the number of days since the upload; sixth, the view ratio (views/day); seventh, the number of likes; ninth, the number of dislikes; tenth, the like ratio (like*100/like+dislike); and eleventh, the video power index (VPI). Video popularity is indicated by the VPI, which provides a relative measure of how much a video was liked in relation to the view ratio [33].

Sources of videos uploaded

One reviewer chose a primary source to help with categorization when a video could be found in more than one of these sources; a second reviewer verified the accuracy of 25 randomly chosen videos. The following categories were used for organizing video sources: academic (referring to authors or uploaders connected to academic institutions or research teams), physicians (individual doctors or doctor groups without affiliations to universities or colleges or research affiliations), non-physicians or medical professionals who are not licensed physicians, actual exercise equipment, medical resources (animations or content from websites related to health), individuals, business-related (commercial) and other (videos that did not belong to the aforementioned sources). 

Video material

A primary content type was chosen by one reviewer to help with categorization when a video could fit into multiple categories; a second reviewer verified the accuracy of a random 25% of these videos. One of the following categories applied to the primary video content: self-breast examination, advertisements, disease-specific information, and patient experience.

Reliability of videos and the quality of educational material

The quality of educational content and the dependability of videos were evaluated by applying benchmark criteria from the Journal of the American Medical Association (JAMA) [34]. This non-specific tool objectively evaluates four criteria: authorship, attribution, currency, and disclosure (Table 1). Each criterion met is worth one point, with a possible total score of four. The validity of YouTube videos has previously been assessed using this scoring system in academic publications [32,35].

**Table 1:** The JAMA criteria and descriptions used for rating video reliability Source: Silberg et al. [34]

Criteria	Description
Authorship	Author and contributor credentials and affiliations are clearly stated
Attribution	Clearly lists all copyright information and includes references or sources for content
Currency	Date of post and subsequent updates to content are included
Disclosure	Conflicts of interest, funding, sponsorship, advertising, support, and video ownership are disclosed

The general educational content of the videos was evaluated using the Global Quality Score (GQS) [36]. The GQS is a tool for ranking educational resources from poor to excellent quality, with a score range of one to five, with five indicating the highest educational quality (Table 2).

**Table 2:** The GQS grades and descriptions for rating video educational quality Source: Bernard et al. [36]

Grade	Description
1	Poor quality; not useful for patient education.
2	Poor quality; minimal relevant information. Limited utility to patients.
3	Suboptimal quality; some useful information present, but missing key topics. Somewhat useful to patients.
4	Good quality; most important topics discussed. Useful to patients.
5	Excellent quality; all topics covered in a clear manner. Highly useful to patients.

A nine-item scale was created based on the instructions found in the National Guidelines for Self-breast Examination and Clinical Breast Examination [37]. This score assessed the video comprehensiveness for BSE-specific instructions in which a point is awarded for meeting each criterion, with a maximum score of nine (Table 3). 

**Table 3:** National guidelines for self-breast examination and clinical breast examination Source: Gulve et al. [37]

Criteria
The proper timing to conduct BSE is stated (1 week after menstrual cycle)
The proper settings of examination are explained (Chest exposure, examining the breasts in front of a mirror with hands on hip)
Instruction to examine the breast with raised hands
Instruction to look for breast size and symmetry
Instruction to look for breast skin changes (Color change, bulging, dimpling)
Instruction to look for breast ulceration
Instruction to examine the nipples’ shape and discharge
Instruction about breast palpation technique
Instruction to feel the breast while lying down

Statistical analysis

The mean and standard deviation were used for the descriptive analysis of metric variables, while frequency and proportion (%) were given for categorical variables. A one-way ANOVA test was conducted to determine the mean differences between JAMA, BSE, and video quality scores in relation to video sources and the type of video content. All analyses were performed using the software program Statistical Packages for Software Sciences (SPSS) version 21 (IBM Inc., Armonk, New York).

## Results

Table 4 presents the characteristics of the analyzed videos. The mean video duration was 3.71 minutes. The mean frequency of total video views was 311,625.9, with mean daily views of 199.6. The mean number of days since the upload was 1524.6. The mean frequency of likes, dislikes, and like ratio were 3848.7, 346.7, and 87.9, respectively. Additionally, the mean VPI was 184.2.

**Table 4:** Characteristics of the 50 analyzed videos

Variables	Mean ± SD
Video duration in minutes	3.71 ± 2.95
Number of views	311625.9 ± 1000187.9
Days since upload	1524.6 ± 1077.9
View ratio (views/day)	199.6 ± 517.9
Number of likes	3848.7 ± 11208.9
Number of dislikes	346.7 ± 1974.9
Like ratio	87.9 ± 16.7
Video power index	184.2 ± 489.1

Regarding the upload sources, the most common was medical sources (30 videos, 60%), followed by commercial sources (eight videos; 16%), physicians (seven videos, 14%), and multiple sources (three videos, 6%), whereas the least common was academic sources (two videos, 4%; Table 5).

**Table 5:** Video upload sources

Sources	Number of videos (%)
Medical source	30 (60%)
Commercial	8 (16%)
Physician	7 (14%)
Academic	2 (4%)
Multiple source	3 (6%)

**Figure 1:**
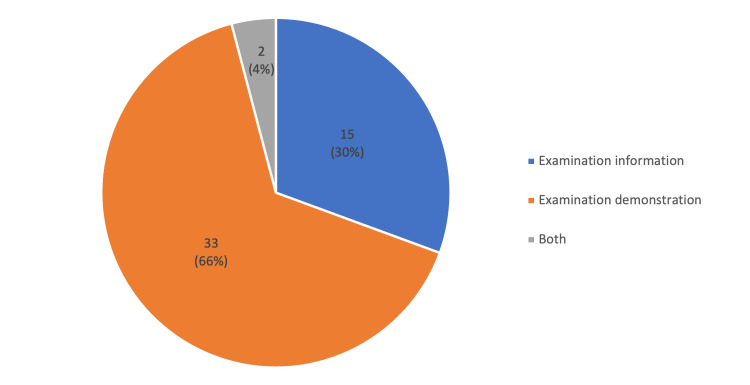
Type of content

The content of the included videos was mainly examination demonstration (33 videos, 66%), while 15 of the videos (30%) contained examination information. Only two videos (4%) displayed both examination information and demonstration (Figure 1).

Regarding the video reliability using the JAMA benchmark criteria, 17 and 19 videos scored 0 and 1, respectively, constituting 72% of the assessed videos. Only one video (2%) scored full marks for reliability (Table 6).

**Table 6:** Video reliability using the JAMA benchmark criteria JAMA - Journal of the American Medical Association

JAMA score	Number of videos (%)
0	17 (34%)
1	19 (38%)
2	12 (24%)
3	1 (2%)
4	1 (2%)

The GQS score for video quality showed that only four videos (8%) scored 5/5 (excellent quality), while 16 (32%) and 20 (40%) videos were considered of good and suboptimal quality, respectively. However, 10 (20%) videos were of poor quality (Table 7).

**Table 7:** Global Quality Score for videos educational quality assessment GQS - Global Quality Score

GQS score	Number of videos (%)
Poor quality; is unlikely be to use for patient education	0 (0%)
Poor quality; is of limited use to patients because only some information is present.	10 (20%)
Suboptimal quality and flow; is somewhat useful to patients; important topics are missing, some information is present	20 (40%)
Good quality and flow; useful to patients because most important topics are covered	16 (32%)
Excellent quality and flow; is highly useful to patients	04 (8%)

Table 8 presents the video educational quality using comprehensiveness scores out of the nine items specific to self-breast screening, according to the referenced guidelines. The most common scores were eight and nine equally, with 17 videos for each score. This constitutes 68% of the assessed videos. However, four videos (8%) scored 0.

**Table 8:** Assessment of video educational quality based on BSE comprehensiveness score BSE - breast self-examination

Number of BSE based on guidelines	Number of videos (%)
0	4 (8%)
1	0 (0%)
2	0 (0%)
3	1 (2%)
4	4 (8%)
5	0 (0%)
6	6 (12%)
7	1 (2%)
8	17 (34%)
9	17 (34%)

Overall, the mean scores for JAMA, GQS, and BSE comprehensiveness were 1.00, 3.24, and 7.02, respectively. Furthermore, based on the one-way ANOVA test, we noted that no significant differences existed between the upload sources in relation to the JAMA score (p=0.078), GQS score (p=0.899), and BSE guidelines score (p=0.968). However, we found significant differences between the video content of both examination information and demonstration in terms of video GQS score (p=0.002) and BSE comprehensiveness score (p=0.002; Table 9).

**Table 9:** Videos reliability and educational quality stratified by upload source and video content JAMA - Journal of the American Medical Association; BSE - breast self-examination ** Significant at p<0.05 level

Variables	N	Mean JAMA score ± SD	Mean video quality score ± SD	Mean BSE Guidelines score ± SD
Overall	50	1.00 ± 0.93	3.24 ± 0.98	7.02 ± 2.63
Upload source				
Medical source	30	0.87 ± 0.82	3.27 ± 1.01	6.80 ± 3.11
Commercial	08	1.25 ± 1.04	3.25 ± 0.89	7.50 ± 1.93
Physician	07	1.14 ± 0.69	3.00 ± 0.82	7.29 ± 1.25
Academic	02	2.50 ± 2.12	3.00 ± 1.41	7.00 ± 1.41
Multiple source	03	0.33 ± 0.58	3.67 ± 1.53	7.33 ± 2.89
Video content				
Examination information	15	0.93 ± 1.03	2.60 ± 1.06	5.13 ± 3.76
Examination demonstration	32	1.03 ± 0.89	3.47 ± 0.80	7.75 ± 1.41
Both	02	1.50 ± 0.71	4.50 ± 0.71 **	9.00 ± 0.00 **

## Discussion

BSE has a crucial role in early breast cancer detection, which promotes improved survival. However, an appropriate application and understanding of the concerning findings are required to guide medical counseling. Thus, education is important in its effectiveness. Using YouTube may enhance public education on this subject [38]. Thus, we aimed to determine the quality and reliability of YouTube videos about BSE and video features associated with higher educational quality.

We observed that YouTube videos on BSE were relatively attractive. This was evident by more daily views compared to other reports with similar concepts [39,40]. The average number of daily views was nearly 200, with mean total views of 311,625.9 ± 1,000,187.9. Medical sources were the predominant sources of the uploaded videos (30 videos, 60%), followed by commercial sources (eight videos, 16%), physicians (seven videos, 14%), and multiple sources (three videos, 6%), and academic sources (two videos, 4%). Although medical sources were previously correlated with the best reliability and quality in BSE videos [41,42], they did not show a significant contribution to video quality or reliability in the current study. However, academic sources showed the highest reliability and quality scores. Although videos created by physicians have been suggested to be more reliable and better quality [43], we found contradicting results. This finding is comparable to other reports [32,42].

Examination demonstration was the most prevalent video content (33 videos, 66%), while only two videos (4%) demonstrated both examination information and demonstration. Despite being the least prevalent content type, this showed significantly enhanced quality scores. Conversely, videos containing only examination information were of the lowest quality and reliability.

In 2018, Esen et al. [41] conducted a study to examine the quality and reliability of YouTube videos related to BSE. They found that 62% of YouTube videos about BSE were unreliable, had low GQS scores, and lacked demonstration of the proper elements of BSE. In the current study, 34% of videos (17 videos) scored 0, 38% (19 videos) scored one, and only 2% (one video) scored four in accordance with the JAMA reliability score. This demonstrates that most of the videos were poor in reliability and integrity. This finding is consistent with previous studies [32,44]. Furthermore, we found that most of the examined videos were unreliable even when stratified by the upload source and video content. Regarding the GQS score, only 8% (four videos) demonstrated excellent quality, whereas 32% (16 videos) were considered good quality. However, the majority (30 videos, 60%) were within poor to suboptimal categories. This reflects the wide variation of educational quality scores for BSE videos on YouTube, with more toward lower scores. Conversely, 35 of the analyzed videos (70%) covered seven to nine of the nine points required for adequate BSE. These high comprehensiveness scores show that most of the videos illustrated the proper instructions specific to BSE, making them relatively informative. However, nine videos (18%) had very low scores (0-4/9). Although they were scarce, some of them were found among the top 20 relevant videos on BSE on YouTube, raising concerns about the impact of this misleading content on the public.

The study findings have important implications for developing more reliable and comprehensive videos to ensure educational quality. Thus, it is necessary for academic and medical institutions to collaborate in order to generate trustworthy educational content and to fullfil their role in enhancing public education in a way that is beneficial. Furthermore, We recommend that there should be a greater number of academic martial videos aimed at educating the general public about various preventative measures. To reach everyone who needs assistance, these should adhere to particular updated guidelines and be in a variety of languages.

A limitation of our study is that only English-language videos were assessed. Furthermore, video inclusion was limited to the most popular and restricted to 50 videos. Despite these videos being more likely to be found by viewers, they may not reflect the entire YouTube content of the study object.

## Conclusions

YouTube videos of BSE are attracting many viewers. Videos containing examination information and demonstrations showed the best educational quality. Although most of the YouTube videos of BSE showed a high comprehensiveness score for BSE-specific instructions, their JAMA reliability and GQS scores were poor.

The reliability and educational quality of the videos must be improved to use this platform efficiently. This requires synergy between academic and medical institutes to produce reliable educational material and activate their role in usefully enriching public education.
